# Comparative analysis of circular RNAs between soybean cytoplasmic male-sterile line NJCMS1A and its maintainer NJCMS1B by high-throughput sequencing

**DOI:** 10.1186/s12864-018-5054-6

**Published:** 2018-09-12

**Authors:** Linfeng Chen, Xianlong Ding, Hao Zhang, Tingting He, Yanwei Li, Tanliu Wang, Xiaoqiang Li, Ling Jin, Qijian Song, Shouping Yang, Junyi Gai

**Affiliations:** 1 Soybean Research Institute, National Center for Soybean Improvement, Key Laboratory of Biology and Genetic Improvement of Soybean (General, Ministry of Agriculture), State Key Laboratory of Crop Genetics and Germplasm Enhancement, Jiangsu Collaborative Innovation Center for Modern Crop Production, Nanjing Agricultural University, Nanjing, 210095 China; 2 Soybean Genomics and Improvement Laboratory, Beltsville Agricultural Research Center, USDA-ARS, Beltsville, MD 20705 USA

**Keywords:** Soybean (*Glycine max* (L.) Merr.), Cytoplasmic male sterility, CircRNAs, High-throughput sequencing, Parental genes, Binding miRNAs

## Abstract

**Background:**

Cytoplasmic male sterility (CMS) is a natural phenomenon of pollen abortion caused by the interaction between cytoplasmic genes and nuclear genes. CMS is a simple and effective pollination control system, and plays an important role in crop heterosis utilization. Circular RNAs (circRNAs) are a vital type of non-coding RNAs, which play crucial roles in microRNAs (miRNAs) function and post-transcription control. To explore the expression profile and possible functions of circRNAs in the soybean CMS line NJCMS1A and its maintainer NJCMS1B, high-throughput deep sequencing coupled with RNase R enrichment strategy was conducted.

**Results:**

CircRNA libraries were constructed from flower buds of NJCMS1A and its maintainer NJCMS1B with three biological replicates. A total of 2867 circRNAs were identified, with 1009 circRNAs differentially expressed between NJCMS1A and NJCMS1B based on analysis of high-throughput sequencing. Of the 12 randomly selected circRNAs with different expression levels, 10 showed consistent expression patterns based on high-throughput sequencing and quantitative real-time PCR analyses. Tissue specific expression patterns were also verified with two circRNAs by quantitative real-time PCR. Most parental genes of differentially expressed circRNAs were mainly involved in biological processes such as metabolic process, biological regulation, and reproductive process. Moreover, 83 miRNAs were predicted from the differentially expressed circRNAs, some of which were strongly related to pollen development and male fertility; The functions of miRNA targets were analyzed using Gene Ontology (GO) and Kyoto Encyclopedia of Genes and Genomes (KEGG), and the target mRNAs were significantly enriched in signal transduction and programmed cell death. Furthermore, a total of 165 soybean circRNAs were predicted to contain at least one internal ribosome entry site (IRES) element and an open reading frame, indicating their potential to encode polypeptides or proteins.

**Conclusions:**

Our study indicated that the circRNAs might participate in the regulation of flower and pollen development, which could provide a new insight into the molecular mechanisms of CMS in soybean.

**Electronic supplementary material:**

The online version of this article (10.1186/s12864-018-5054-6) contains supplementary material, which is available to authorized users.

## Background

Soybean (*Glycine max* (L.) Merr.) is an important oil and protein crop, but soybean yield is low. Utilization of heterosis has been proved to be one of the effective methods to increase crop yield, and cytoplasmic male sterility (CMS) plays an important role in heterosis utilization [1]. CMS is a maternally inherited phenotype caused by the interaction of cytoplasmic genes and nuclear genes, which lead to pollen abortion but normal pistil development [2, 3]. To date, CMS has been observed in over 200 plant species [4] and is often obtained in wild germplasm or by inter- or intra-subspecies backcrossing [5]. In soybean, CMS was first reported by Davis [6], after which many studies on CMS were conducted [7, 8]. In recent years, transcriptomics [9], proteomics [10], microRNA [11], and DNA methylation of CMS have been explored [12], but the function of circular RNAs (circRNAs) in soybean CMS has not been reported.

CircRNAs are a class of endogenous noncoding RNAs, which do not have 5′ caps and 3′ tails and form a ring structure with covalent bonds [13]. CircRNAs were first reported based on deep sequencing of RNA by Salzman et al. [14], which were neglected for decades because they were considered as transcriptional noise or reverse transcription PCR artifacts by-product [15]. With the development of high-throughput sequencing technology and bioinformatics, circRNAs have been identified in all domains of life, including eukaryotes [15, 16], archaea [17], bacteria [18], and viruses [19]. Previous studies showed that circRNAs were more resistant to degradation by RNase R than their linear counterparts [20], and had tissue, cell-type, and developmental-stage specific expression patterns [15, 21, 22]. Li et al. [23] demonstrated that the circRNAs located in the nucleus could promote transcription of their parental genes via specific RNA-RNA interactions, but another recent study revealed that circRNAs derived from organelle genome could also regulate gene expression [24]. Furthermore, circRNAs act as miRNA sponges and prevent them from inhibiting their target mRNAs [25, 26]. Most recent studies have demonstrated that some circRNAs can be translated into polypeptides or proteins by translation initiation element internal ribosome entry site (IRES) or N6-methyladenosine (m^6^A) [27, 28].

Although not as comprehensive as in animals, the exploration of circRNAs in plants is increasing. Differential expression of circRNAs has been reported in plants, for example, 27 rice exonic circRNAs were associated with phosphate starvation responsive expression [16], 163 tomato circRNAs with chilling responsive expression [29], and 62 wheat circRNAs with dehydration stress specific expression [30]. Moreover, some circRNAs showed time, tissue, species, or developmental-stage specific expression patterns in plants [16, 24, 31, 32]. Unlike the positive regulation of circRNAs in animals [23], over-expression of circRNAs in rice and tomato reduced expression level of their parental genes [33, 34]. In soybean, a total of 5372 circRNAs were identified, of which approximately 80% were generated from paralogous genes [35]. Meanwhile, Zhao et al. [35] found that up-regulation of circRNAs might decrease the activity of target miRNAs and increase expression of the related mRNAs.

To explore the expression profile and possible functions of circRNAs in the soybean CMS line NJCMS1A vs. its maintainer line NJCMS1B, high-throughput deep sequencing coupled with RNase R enrichment strategy was conducted. Target miRNAs of differentially expressed circRNAs and the correlated mRNAs were predicted using bioinformatics methods, and their potential functions were further analyzed. Our study investigated the possible role of circRNAs in CMS for the first time, and the results showed that the circRNAs might participate in the regulation of flower and pollen development in soybean.

## Methods

### Plant materials and sample collection

The soybean cytoplasmic male-sterile line NJCMS1A was developed through consecutive backcross. The cultivar N8855 was the donor and cultivar N2899 (designated as NJCMS1B afterwards) was a recurrent parent [8, 36, 37]. NJCMS1A and NJCMS1B are near-isogenic lines of isonuclear alloplasmic type with similar nucleus but different cytoplasm. NJCMS1A and NJCMS1B were grown in the summer of 2016 at Dangtu Experimental Station, National Center for Soybean Improvement, Nanjing Agricultural University, Maanshan, Anhui, China. Male sterile plants were identified by observing dehiscence of anthers, germination rate of pollen, and morphological traits of plants at maturity. Because it is difficult to determine development stage of pollen based on flower bud size in soybean, during the flowering period, the flower buds of different sizes were collected from NJCMS1A or NJCMS1B plants and mixed, with three biological replicates per line. Meanwhile, roots, stems and leaves of soybean were also collected at the flowering period with three biological replicates. The tissues were immediately frozen in liquid nitrogen and stored at − 80 °C for further use.

### Total RNA extraction and RNase R treatment

Total RNA from flower buds of NJCMS1A and NJCMS1B lines was extracted using the RNAprep pure Plant Kit (Qiagen, DEU) according to the manufacturer’s protocol, DNA contamination was removed using the DNase I contained in the Kit. For RNase R-treated total RNA samples, the purified DNaseI-treated total RNA was incubated for 15 min at 37 °C with 3 units RNase R per μg of total RNA (Epicentre, Shanghai, CN). RNA was subsequently purified using an RNase MinElute Cleaning Kit (Qiagen, DEU). To obtain accurate and sufficient transcriptome data, RNA from three biological replicates of NJCMS1A and NJCMS1B lines were sequenced.

### CircRNA library construction and sequencing

rRNA-depleted RNAs in 5 μg RNA per sample was obtained using the Epicentre Ribo-zero™ rRNA Removal Kit (Epicentre, USA) and were further treated with RNase R (Epicentre, USA) for Trizol extraction. Sequencing libraries were prepared using NEBNext Ultra Directional RNA LibraryPrep Kit (NEB, USA) following manufacturer’s instructions. The libraries were preliminarily quantified by Qubit and diluted to 1 ng/ul. The insert size of the libraries was detected by Agilent 2100/Caliper, which was expected to be distributed around 250~ 300 bp. The effective concentration of the libraries was accurately quantified by qRT-PCR, and the effective concentration was greater than 2 nM to ensure the libraries quality. Briefly, fragmentation was carried out using divalent cations under elevated temperature in NEBNext First-strand Synthesis Reaction Buffer. First-strand cDNA was synthesized using random hexamer primer and M-MuLV reverse transcriptase (RNaseH). Second-strand cDNA synthesis was subsequently performed using DNA Polymerase I and RNase H. In the reaction buffer, dTTP in dNTPs was replaced by dUTP. Remaining overhangs were blunted via exonuclease/polymerase. After adenylation of 3′ ends of DNA fragments, NEBNext Adaptor with hairpin loop structure was ligated for hybridization. In order to obtain cDNA fragments of 150–200 bp in length, the library fragments were purified with the AMPure XP system (Beckman Coulter, Beverly, USA). Subsequently, 3 μl USER Enzyme (NEB, USA) was added to adaptor-ligated cDNA of 150–200 bp and kept at 37 °C for 15 min followed by 5 min at 95 °C before PCR. PCR was performed with Phusion high-fidelity DNA polymerase, universal PCR primer, and index (X) primer. Finally, the library was purified (AMPure XP system) and then qualified by the Agilent Bioanalyzer 2100 system. Clustering of the index-coded samples was performed on a cBot Cluster Generation System using HiSeq PE Cluster Kit v4 cBot (Illumina) according to the manufacturer’s instructions. After cluster generation, the libraries were sequenced on an Illumina Hiseq 2500 platform and 125 bp paired-end reads were generated. Library construction and sequencing were carried out by Novogene (Novogene, Beijing, China).

### Identification of circRNAs and differential expression analysis

Raw data (raw reads in fastq format) was first processed by a custom perl script. Sequence reads with adapter, ploy-N, and low-quality were eliminated. The remaining reads were used for read quality and GC content estimation, and downstream calculation. Soybean reference genome and gene annotation were downloaded from the website at https://phytozome.jgi.doe.gov/pz/portal.html#. The index of the reference genome was built using Bowtie v2.0.6, paired-end reads were aligned to the reference genome (soybean genome version 2.0 in Phytozome) using TopHat v2.0.9. 20-mers from 5′ and 3′ end of the unmapped reads were extracted and aligned independently to reference sequences using Bowtie v2.0.6. Anchor sequences were extended by find_circ [15], such that the complete read was aligned and the breakpoints were flanked by GU/AG splice sites. Subsequently, the back-spliced reads with at least two supporting reads were annotated as circRNAs.

Expression level of circRNAs was normalized by transcript per million (TPM) through the following criteria [38]: Normalized expression = (mapped reads)/(total reads) * 1000000. Differential expression between two groups was performed using DESeq2 (version 1.6.3) [39]. Differentially expressed circRNAs were identified with the cutoff threshold of |log_2_ (fold-changes)| ≥ 2 based on the method used by Wang et al. [30] and Liu et al. [31].

### Prediction of miRNA targets of circRNAs, mRNA targets of miRNA, and annotation of functions

miRNA binding sites of differentially expressed circRNAs were predicted by psRobot_tar in psRobot [40]. Meanwhile, the circRNA-miRNA interaction network was delineated by Cytoscape [41]. The obtained miRNAs were used to predict the target mRNAs by psRobot with default parameters [40]. The parental genes of differentially expressed circRNAs and the predicted target mRNAs were classified into different functional processes based on Gene Ontology (GO) term enrichment using the Web Gene Ontology Annotation Plot (WEGO) [42] and agriGO [43, 44], respectively. The KOBAS [45] was used for KEGG pathways enrichment analysis. The gene annotation of *A. thaliana* at http://www.*arabidopsis*.org/ was used to define functions of homologous genes in soybean.

### PCR amplification and sanger sequencing

To validate the soybean circRNAs identified in this study, a set of 12 differentially expressed circRNAs were amplified with divergent primer. As a control, a pair of convergent primers were designed for gma-circRNA0002 (Additional file 1). All primers were synthesized by Invitrogen (Shanghai, China). The total and RNase R-treated RNA of each sample were used as templates. PCR products were separated using agarose gel, and each band was excised and purified using the AxyPrep DNA Gel Extraction Kit (Axygen, Suzhou, China) for Sanger-sequencing.

### Quantitative real-time PCR validation

Quantitative real-time PCR (qRT-PCR) was carried out to validate differential expressional levels of circRNAs. Divergent primers were designed in order to obtain amplicon from circle template (Additional file 1). According to the instruction of the iScript Select cDNA Synthesis Kit (BIO-RAD, USA), 1 μg of the total RNA untreated with RNase R was reverse-transcribed with random primers. Expression of circRNAs was quantified using the iTaq Universal SYBR Green Supermix (BIO-RAD, USA) on the Bio-Rad CFX96 machine (CFX96 Touch, BIO-RAD, USA). All real-time PCR assays were performed with three biological replicates, and the expression of the housekeeping gene *GADPH* was used as a reference for data normalization. The qRT-PCR aliquot contained 2 μL cDNA, 0.6 μL of each upstream and downstream primers (10 μM), 10 μL Takara SYBR Premix Ex Taq, and 6.8 μL RNase-free ddH_2_O and performed with an initial denaturation at 95 °C for 30 s, followed by 40 cycles at 95 °C for 5 s, 60 °C for 30 s. The amplification curve and melting curve were examined to evaluate specific amplification. The circRNAs relative expression levels (log_2_ values) were calculated using the 2^–ΔΔCt^ method. Student’s t-test was performed to compare differences of circRNAs expression between NJCMS1A and NJCMS1B. The probability threshold of significance was *P* < 0.05.

### Protein-coding potential prediction of circRNAs

The IRES element is required to initiate translation of a mRNA sequence without a 5′-cap structure [46]. If a circRNA has at least one IRES element, it may be able to encode a protein. To predict the IRES elements in soybean circRNAs, we blasted sequences of the circRNAs to all the IRES sequences in the website (http://iresite.org/) at an E-value < 0.05 [47]. To predict the ORFs of predictive circRNAs, we used the prediction algorithm at the website (https://github.com/kadenerlab/cORF_pipeline). Briefly, the longest ORF spanning the circRNA junction was selected for further analysis. The possible coding products of the circRNAs with protein-coding potential were used to predict the conserved domains using the Conserved Domain Database (https://www.ncbi.nlm.nih.gov/Structure/cdd/wrpsb.cgi).

## Results and discussion

### Identification of circRNAs in soybean

Total amount of sequence for each RNA libraries was ≥9 Gb with Q20 ≥ 93%, Q30 ≥ 85%, and an error rate ≤ 0.05 (Table 1), the depth and accuracy of high quality sequence was sufficient for subsequent analysis.

**Table 1:** Summary of circRNA sequencing data

Sample	Replicates	Number of raw reads	Number of clean reads	Clean bases (Gb)	Error rate (100%)	Q20 (100%)	Q30 (100%)	GC content (100%)
	NJCMS1A-1	84,474,228	80,699,898	12.1	0.05	93.38	85.41	54.16
NJCMS1A	NJCMS1A-2	67,528,014	63,913,764	9.58	0.02	97.85	94.07	53.75
	NJCMS1A-3	98,550,080	92,713,788	13.9	0.03	97.29	93.12	54.55
	NJCMS1B-1	74,471,198	68,964,198	10.34	0.03	97.39	93.08	53.11
NJCMS1B	NJCMS1B-2	77,162,554	70,527,006	10.58	0.03	97.04	93.03	53.94
	NJCMS1B-3	88,067,914	84,489,922	12.68	0.05	93.53	85.55	54.67

**Fig. 1:**
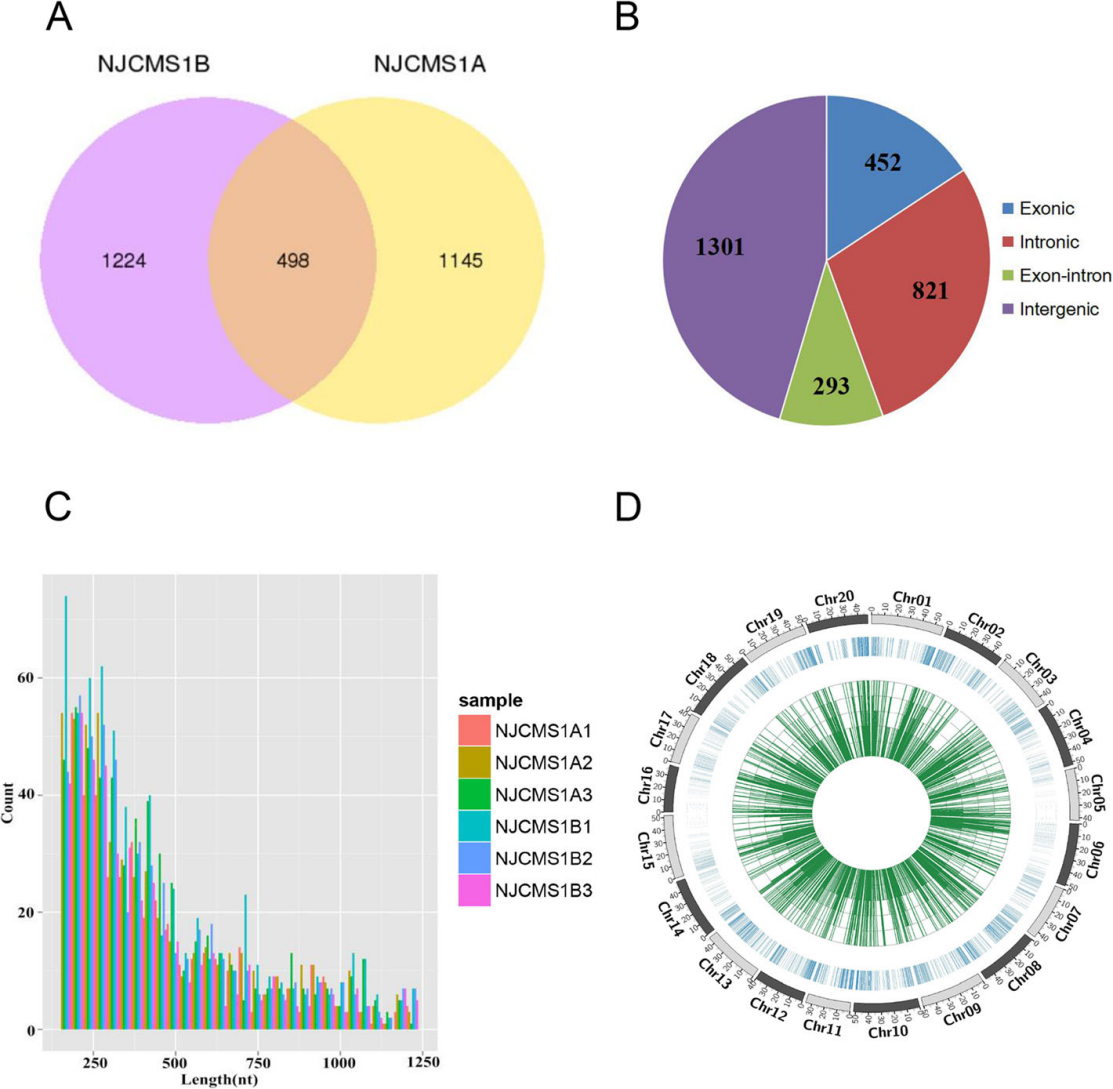
CircRNA sequencing data. (**a**) Venn diagram shows the number of the identified circRNAs in NJCMS1A and NJCMS1B. (**b**) Source statistics of the circRNAs. (**c**) Sequence length distribution of the circRNAs in different samples. (**d**) Circle plot shows the distribution of the identified circRNAs in soybean chromosomes and their expression levels. The outermost layer represents all soybean chromosomes (Chr01-Chr20). The middle blue lines show the distribution of the circRNAs in soybean chromosomes, while the denser lines indicate more circRNA distribution. The innermost green lines show expression levels of the circRNAs, and the height of the lines indicates the level of expression

A total of 2867 circRNAs were identified, which included 1722 from NJCMS1B and 1643 from NJCMS1A (Fig. 1a, Additional file 2). Among them, 452 (15.8%) were derived from exons of a single protein-coding gene (exonic circRNAs), 821 (28.6%) from introns (intronic circRNAs), 293 (10.2%) from exons and introns of one or more genes (exon-intron circRNAs), and 1301 (45.4%) from intergenic regions (intergenic circRNAs) (Fig. 1b). CircRNAs primarily derived from exons in several plants have been reported, e.g. 5152 (85.7%) circRNAs in *A. thaliana* [16], 615 (72.0%) in tomato [29], and 1453 (93.7%) in maize [48]. However, only 2494 (46.4%) exonic circRNAs were identified in soybean by Zhao et al. [35], which was similar to our result. The length of circRNAs in this study ranged from 150 to 44,756 bp, but most (92.8%) were < 2000 bp (Fig. 1c). The length distribution of soybean circRNAs in this study was similar to that reported by Zhao et al. [35]. Additionally, the sequence alignment between the circRNAs inform this study and previous study (Zhao et al. [35]) using blastn indicated that only 78 circRNAs were homologous (Additional file 3) at e-value <1e^− 5^ and identity > 85%. The small proportion of homologous circRNAs in these two studies might be associated with tissue specific expression pattern of circRNA (Additional file 4) and use of different circRNAs prediction software. Besides, the number of circRNAs in different chromosomes and the densities of circRNAs in different chromosomal regions were also different (Fig. 1d).

### Expression profiling of circRNAs and qRT-PCR validation

A total of 1009 circRNAs with at least two-fold (log2) change of expression level between NJCMS1A and NJCMS1B were identified by high-throughput sequencing. Of these, a total of 360 circRNAs were up-regulated, 649 circRNAs were down-regulated in NJCMS1A vs. NJCMS1B (Additional file 5).

**Fig. 2:**
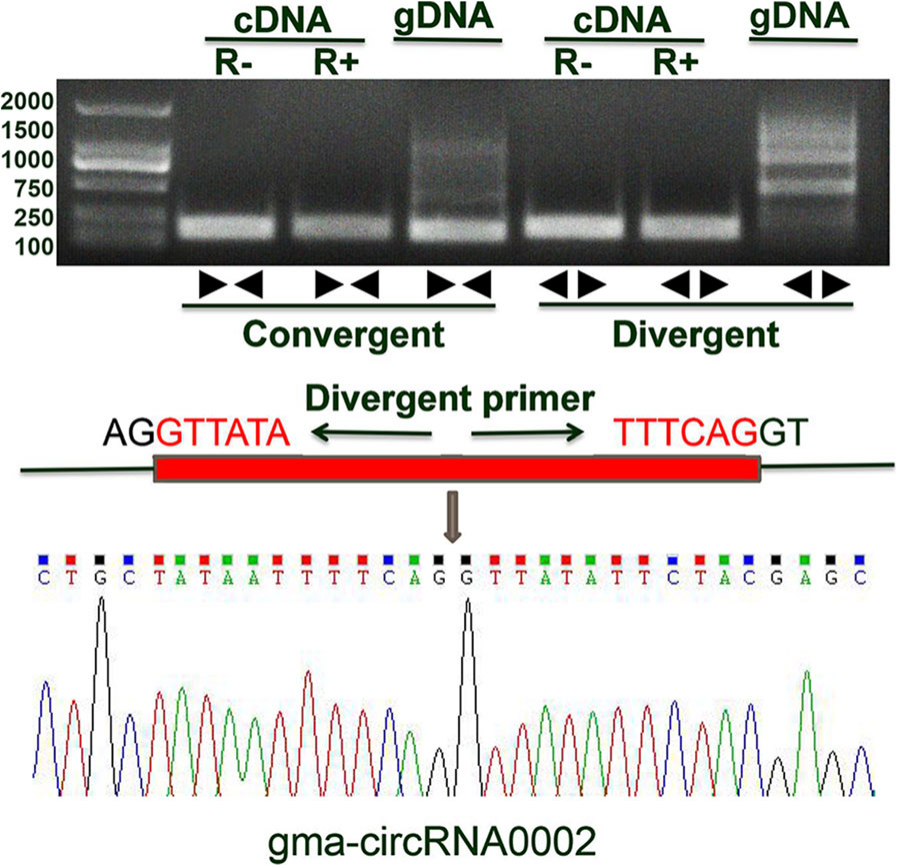
An example of circRNA (gma-circRNA0002) that was validated via amplification and sequencing. R+ and R- represent samples with and without RNase R treatment, respectively

**Fig. 3:**
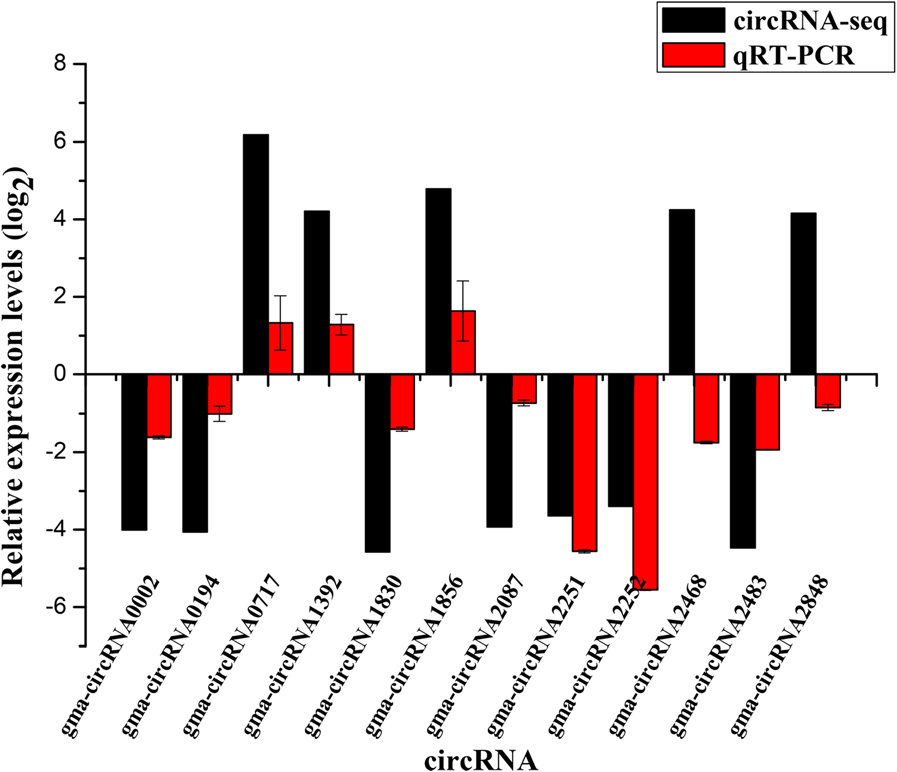
Validation of differentially expressed circRNAs in NJCMS1A and NJCMS1B by qRT-PCR. The x-axis represents the names of circRNAs, while the y-axis represents the circRNA relative expression level generated from qRT-PCR analysis and high-throughput sequencing. Expression of the GADPH gene was used as the internal reference. All qRT-PCR reactions were performed with three biological replicates, and the error bars indicate the standard errors of the means of 2^–ΔΔCt^, with NJCMS1B as a control

The back splicing sites and expression profiles of the 12 randomly selected from 1009 circRNAs were further experimentally validated. We treated total RNA samples with RNase R to verify the special stability (resistance to exonuclease-mediated degradation) of circRNAs and eliminated interference from linear RNA. Divergent primers were designed to guarantee that amplifications were from circular templates and a pair of convergent primers for gma-circRNA0002 were designed as a control. Total RNA samples and RNase R-treated samples were used as reaction templates to amplify circRNAs. As a result, all 12 pairs of divergent primers yielded amplification products with the expected length from cDNAs that with and without RNase R treatment. To demonstrate that the amplification products from the divergent primers were derived from the corresponding circRNAs and spanning the junction sites, we collected the amplification products for further detection by Sanger sequencing (Fig. 2 and Additional file 6). As shown in Fig. 3, among the 12 random-selected circRNAs, 10 showed consistent expression patterns with RNA-seq results. The coincidence rate between qRT-PCR and RNA-seq was 83.3%. Two inconsistent expression of circRNAs candidates (gma-circ2468 and gma-circ2848) may be caused by the low expression levels of the two circRNAs.

### Tissue specific expression patterns validation by qRT-PCR

**Fig. 4:**
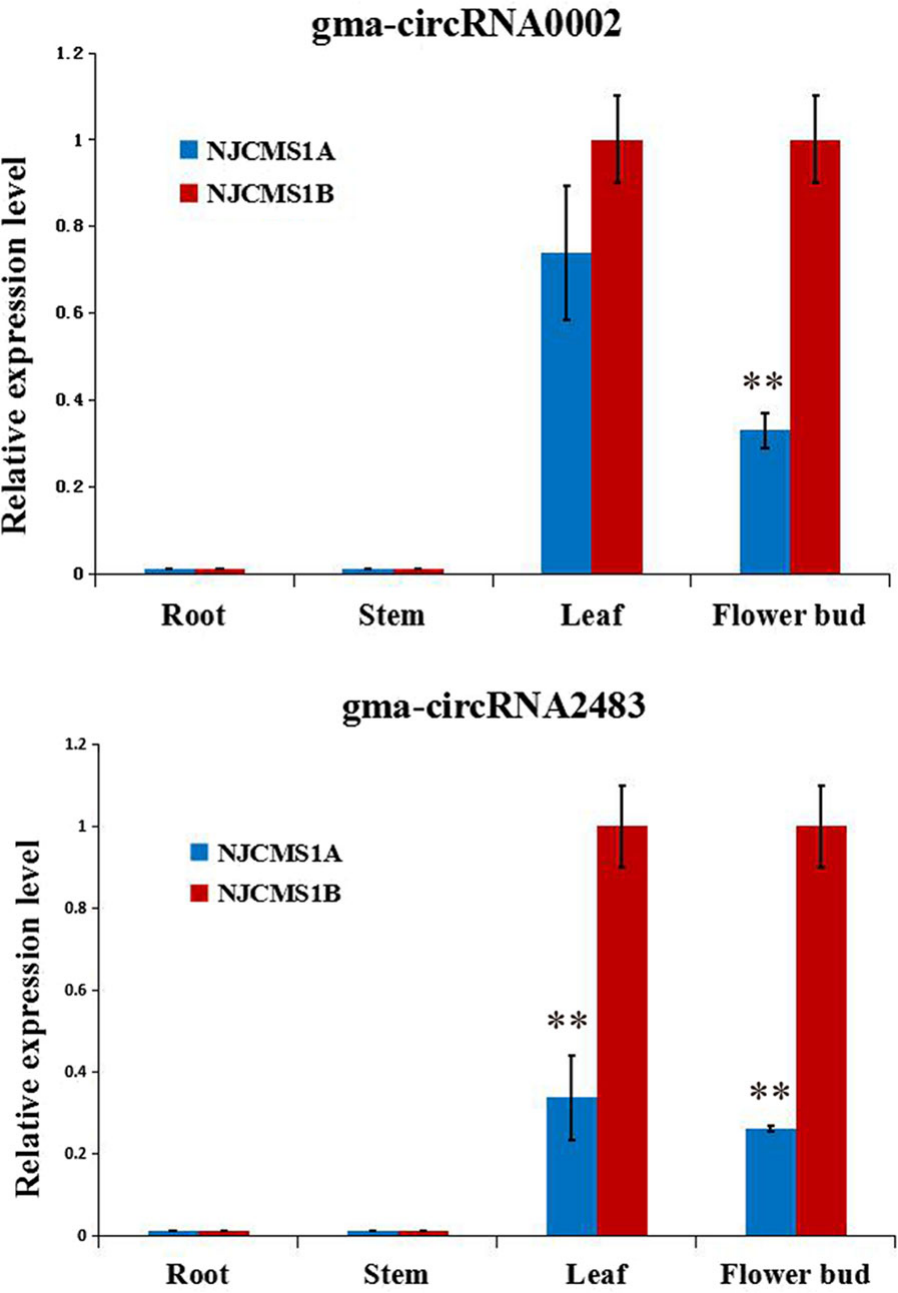
Tissue specific expression patterns validation by qRT-PCR. The relative expression levels of gma-circRNA0002 and gma-circRNA2483 were obtained from roots, stems, leaves and flower buds with three biological replicates by qRT-PCR,, and the error bars indicated the standard error of the mean of 2^–ΔΔCt^, with NJCMS1B as a control. The sign “**” represents *P* < 0.01 according to student t-test, which indicated extremely significantly differences between NJCMS1A and NJCMS1B

Previous studies showed that circRNAs were tissue-preferentially expressed [15, 24]. To verify this, two highly expressed circRNAs (gma-circRNA0002 and gma-circRNA2483) based on the high throughput sequencing were selected for qRT-PCR analysis, these two circRNAs were expressed only in leaves and flower buds of NJCMS1A and NJCMS1B, but not in roots and stems (Fig. 4). The expression level of gma-circRNA0002 was only significantly different in the flower buds, while the expression levels of gma-circRNA2483 were significantly different in the leaves and flower buds between NJCMS1A and NJCMS1B.

### Functional categorization of parental genes of differentially expressed circRNAs

**Fig. 5:**
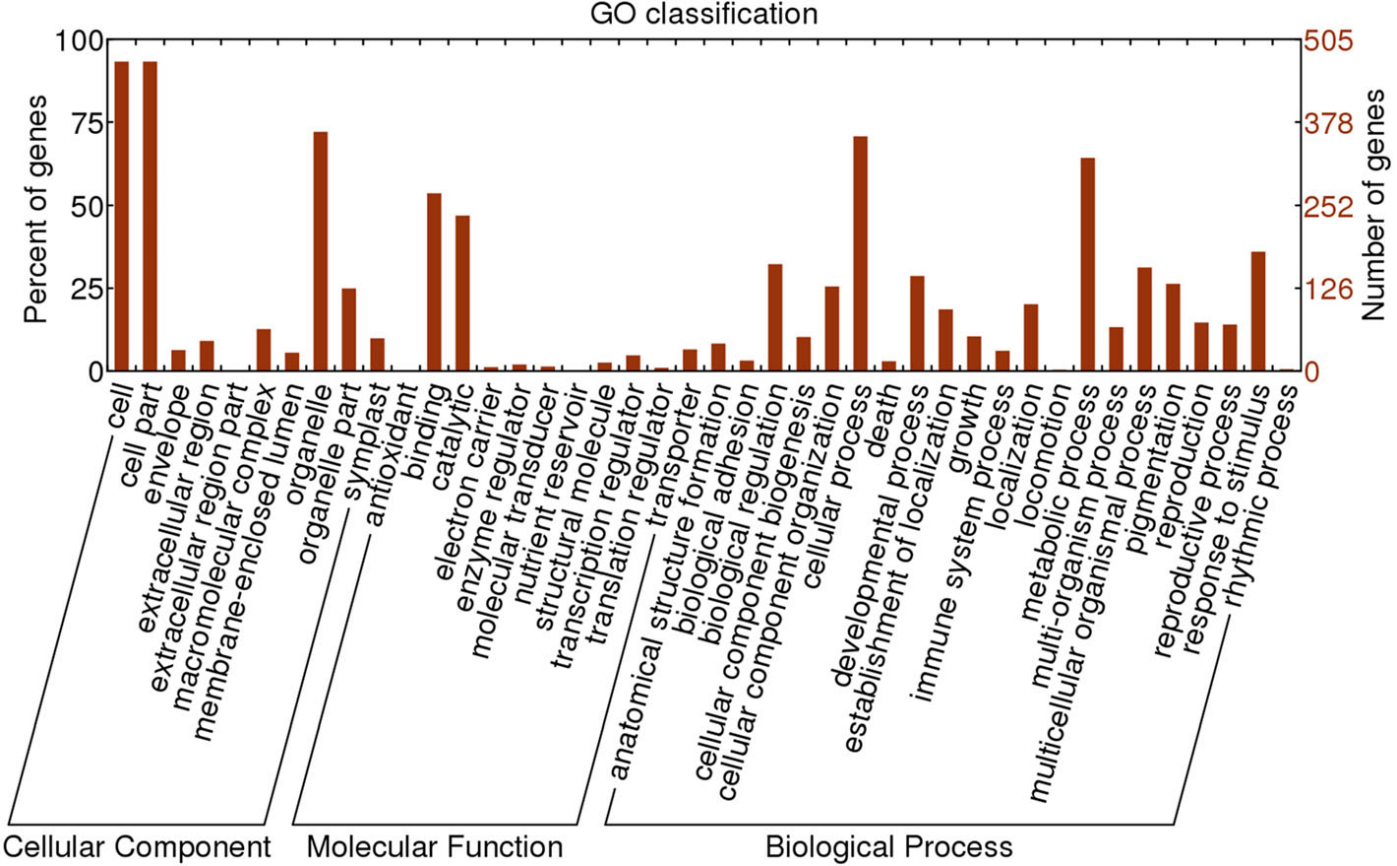
Gene Ontology (GO) annotation of parental genes of the differentially expressed circRNAs between NJCMS1A and NJCMS1B

It has been reported that circRNAs could regulate expression of their parental genes [33, 34]. To understand the possible functions of circRNAs in CMS of soybean, the parental genes of the differentially expressed circRNAs were predicted. A total of 545 parental genes were obtained from the 1009 differentially expressed circRNAs. GO classification showed that these genes are involved in a wide range of biological processes, such as in metabolic process (GO:0008152), biological regulation (GO:0065007), and cellular process (GO:0009987) (Fig. 5). Interestingly, a fraction of parental genes was classified into the categories of reproduction (GO:0000003) and reproductive process (GO:0022414). Among the cellular components, cell (GO:0005623), cell part (GO:0044464), and organelle (GO:0043226) accounted for a large proportion. In the molecular functions, the two main categories were binding (GO:0005488) and catalytic (GO:0003824). GO analysis of the parental genes showed that the differentially expressed circRNAs from NJCNS1A and NJCMS1B were associated with various functions in different biological processes, cellular components, and molecular function, indicating that circRNAs may play an important role in the fertility of soybean. KEGG pathway analysis identified 7 pathways including valine, leucine and isoleucine degradation, selenocompound metabolism, RNA transport, synthesis and degradation of ketone bodies, ascorbate and aldarate metabolism, ubiquitin mediated proteolysis, and porphyrin and chlorophyll metabolism (Additional file 7). These pathways are mainly related to amino acid degradation and material metabolism.

### Putative functions of parental genes of differentially expressed circRNAs in flower development and male fertility

Based on the gene annotation of *A. thaliana*, the function of many parental genes were related to flower development and male fertility. We randomly enumerated 20 parental genes (Table 2) and selected four of them to describe their functions in detail. Glyma.06G173500, the parental gene of gma-circRNA0717, was a homolog of the Glucan Synthase-Like 8 (*GSL8*) gene in *A. thaliana*. *GSL8* and Glucan Synthase-Like 10 (*GSL10*) were two members of the *A. thaliana GSL* gene family, which are independently required for male gametophyte development and plant growth. Experiments showed that *GSL8* and *GSL10* T-DNA insertions led to pollen sterility [49]. *GLS8* was believed to be involved in the synthesis of cell wall component callose [50]. It was hypothesized that gma-circRNA0717 may play a vital role in male gametophyte development. Glyma.13G230500, the parental gene of gma-circRNA1856, was an ortholog of the maternal effect embryo arrest 18 (*MEE18*) gene in *A. thaliana*. Mutation of *MEE18* may affect pollen gametogenesis, pollen germination, pollen tube growth, polarity or guidance, and pollen tube-embryo sac interactions or fertilization [51]. X-ray induced 1 gene (*XRI1*) was a novel DNA repair factor and was essential for male and female meiosis, and homozygous *XRI1* mutants caused complete sterility in *A. thaliana* [52]. In this study, Glyma.18G164900, the parental gene of gma-circRNA2481, was a homolog of the *XRI1* gene. Glyma.18G118100, the parental gene of gma-circRNA2454, was a homolog of squalene epoxidase 1 (*SQE1*) gene in *A. thaliana*. Previous studies showed that *SQE1* mutants displayed severe growth defects in *A. thaliana*, including short stature, short roots, and complete infertility [53]. We speculated that differences in circRNAs expression levels may influence the functions of pollen and male gametophytes, and result in CMS in NJCMS1A by interacting with their parental genes.

**Table 2:** Twenty fertility-related parental genes of differentially expressed circRNAs

circRNAs ID	Parental genes	Homologue Genes (*Arabidopsis thaliana***)**	Function description
gma-circRNA0457	Glyma.04G155600	AT2G24120	male gametophyte development
gma-circRNA0717	Glyma.06G173500	AT2G36850	male gametophyte development; callose component
gma-circRNA1324	Glyma.09G266600	AT3G61050	male gametophyte development; pollen tube growth
gma-circRNA2270	Glyma.16G218700	AT5G22110	male gametophyte development; embryo development
gma-circRNA2801	Glyma.20G205100	AT1G06750	male gametophyte development; pollen tube growth
gma-circRNA0057	Glyma.01G140600	AT1G71820	pollen germination; pollen tube growth
gma-circRNA0685	Glyma.06G135600	AT3G03810	pollen tube development; chloroplast component
gma-circRNA0914	Glyma.07G002300	AT1G78900	pollen development
gma-circRNA1244	Glyma.09G103400	AT2G37270	pollen tube development
gma-circRNA1301	Glyma.09G194900	AT5G65930	pollen germination; plasma membrane component
gma-circRNA1432	Glyma.10G217200	AT1G76850	pollen germination; pollen tube growth
gma-circRNA1856	Glyma.13G230500	AT2G34090	pollen gametogenesis; pollen germination; pollen tube-embryo sac interactions
gma-circRNA2481	Glyma.18G164900	AT5G48720	pollen development; DNA repair; male meiosis
gma-circRNA2791	Glyma.20G182800	AT3G10380	pollen germination; pollen tube growth
gma-circRNA1934	Glyma.14G119800	AT1G43850	flower development; embryo development
gma-circRNA2102	Glyma.15G000300	AT1G16710	flower development; protein amino acid acetylation
gma-circRNA2454	Glyma.18G118100	AT1G58440	flower development; endomembrane system component
gma-circRNA2637	Glyma.19G212600	AT4G02300	flower development; cell wall component
gma-circRNA0464	Glyma.04G163400	AT5G61150	negative regulation of flower development
gma-circRNA2163	Glyma.15G265300	AT1G79280	negative regulation of flower development; stamen development

### Characterization of binding miRNAs of differentially expressed circRNAs

**Fig. 6:**
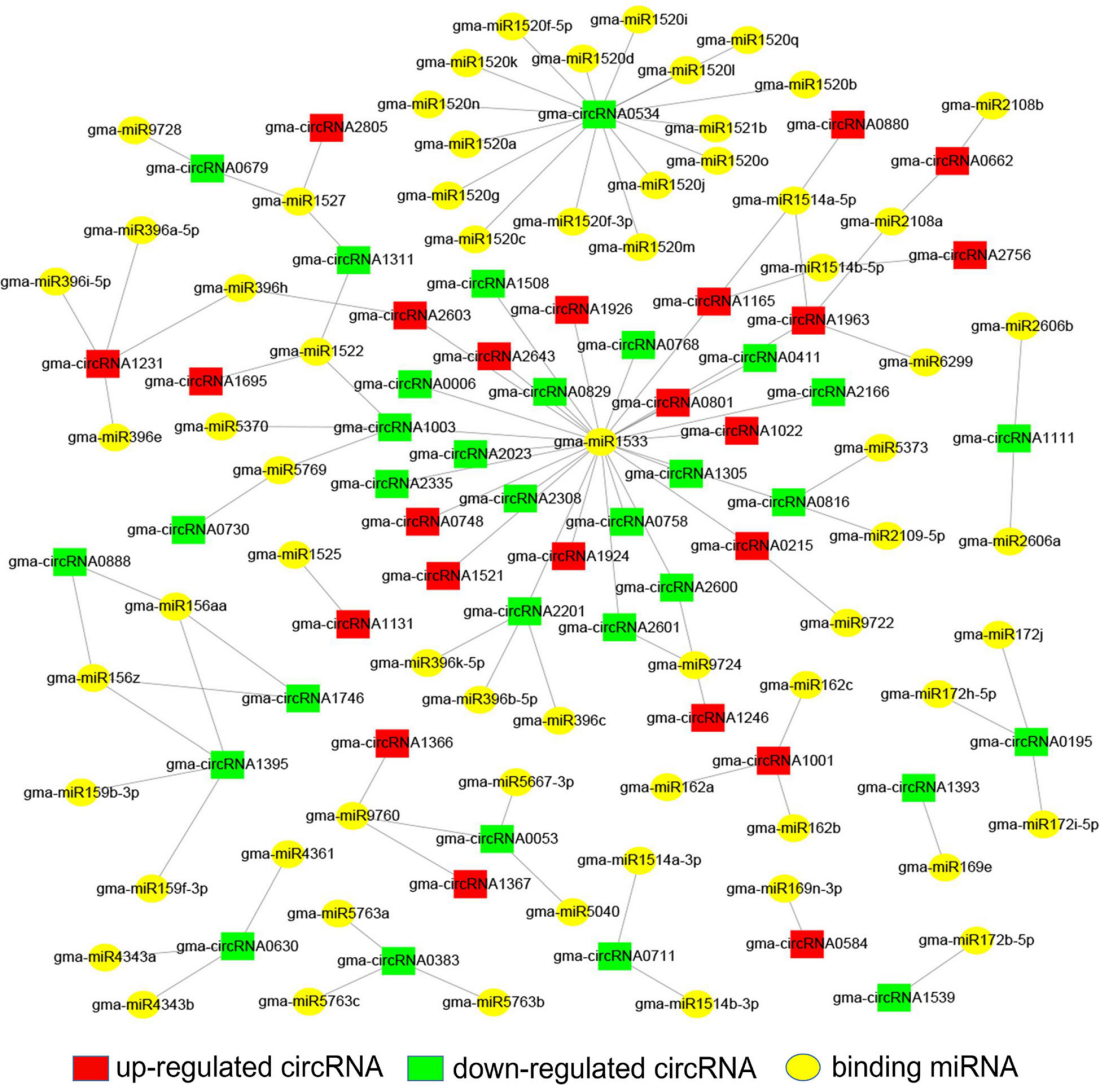
CircRNA-miRNA interaction network for differentially expressed circRNAs in NJCMS1A and NJCMS1B. Red squares represent up-regulated circRNAs, green squares represent down-regulated circRNAs, and yellow circles represent binding miRNAs. The figure comprises 72 differentially expressed circRNAs and their binding miRNAs. Of these circRNAs, 28 are up-regulated and 44 are down-regulated

CircRNAs have been reported to act as miRNA sponges regulating gene expression [25]. To verify if circRNAs have a similar function in soybean, we predicted potential binding sites of miRNAs of the differentially expressed circRNAs. We observed that 72 differentially expressed circRNAs, of which 28 were up-regulated and 44 were down-regulated, contained 83 predicted circRNA-binding miRNAs. Of these circRNAs, only 24 had two to four miRNA binding sites (Additional file 8). Based on the predicted results, a circRNA-miRNA interaction network was delineated by Cytoscape (Fig. 6). The results showed that a single circRNA could target different miRNAs; for example, gma-circRNA0534 targeted to 16 miRNAs. Meanwhile, a single miRNA could be targeted by diverse circRNAs. For instance, gma-miR1533 was targeted by 27 circRNAs.

Among the predicted miRNAs, three miRNAs (gma-miR169e, gma-miR4349, and gma-miR4993) were differentially expressed in our previous study [11]. In addition, we found that some miRNAs, such as miR156, miR162, miR169, and miR172, were important for pollen development and male fertility in soybean. miR156 is one of the highly conserved miRNA families, which was first reported in *A. thaliana* [54]. Squamosa promoter binding like (*SPL*) family proteins are involved in almost all physiological and biochemical processes such as morphogenesis, development stage transition, sporulation, flower and fruit development, external stress response, and hormone signal transduction [55]. Previous studies showed that miR156 regulated the development stage transition [56], flowering process [57], fertility maintenance [58], and fruit ripening [59] in plants by regulating members of the *SPL* family. Gma-circRNA0888, gma-circRNA1395, and gma-circRNA1746 could all target gma-miR156aa and gma-miR156z, which are members of miR156 family and probably involved in flower development. Furthermore, gma-circRNA1001 was predicted to target gma-miR162a, gma-miR162b, and gma-miR162c. NADP-dependent isocitrate dehydrogenase (*NADP-ICDH*) was targeted by gma-miR162a, gma-miR162b, and gma-miR162c according to the degradome analysis performed prior by NJCMS1A and NJCMS1B [11]. *NADPH* is a key cofactor in the cellular redox homeostasis, and is essential in the metabolism of reactive oxygen species (ROS) [60]. In *A. thaliana*, *NADP-ICDH* activity is regulated by molecules involved in ROS, including hydrogen peroxide (H_2_O_2_) [61]. Our previous study showed that these three miRNAs had no significant differences in expression levels between the NJCMS1A and NJCMS1B, but the target gene *NADP-ICDH* was down-regulated in NJCMS1A as revealed by qRT-PCR analysis [11]. The decreased *NADPH* level may result in significant ROS accumulation and male sterility in soybean. Moreover, gma-miR169e was targeted by gma-circRNA1393, and gma-miR169e could target dihydrolipoyl dehydrogenase (*E3*), an important part of the pyruvate dehydrogenase complex. Pyruvate dehydrogenase complex is a critical pathway that supports energy generation for pollen and pollen tube growth [62]. In our previous study, qRT-PCR analysis showed that the target gene *E3* was down-regulated in NJCMS1A [11]. In addition, gma-circRNA0195 was predicted to target gam-miR172j, which could target Glyma.01 g188400, the homolog of the APETALA2 (*AP2*) of *A. thaliana*. In *A. thaliana*, miR172 could control flowering time and floral organ formation by regulating expression of the *AP2*-like transcription factor. A previous study indicated that over-expression of miR172 caused flower development abnormalities, and its phenotype was similar to *ap2* mutants, leading to abnormal gametophyte development [63].

### Functional annotation of predicted target mRNAs

**Fig. 7:**
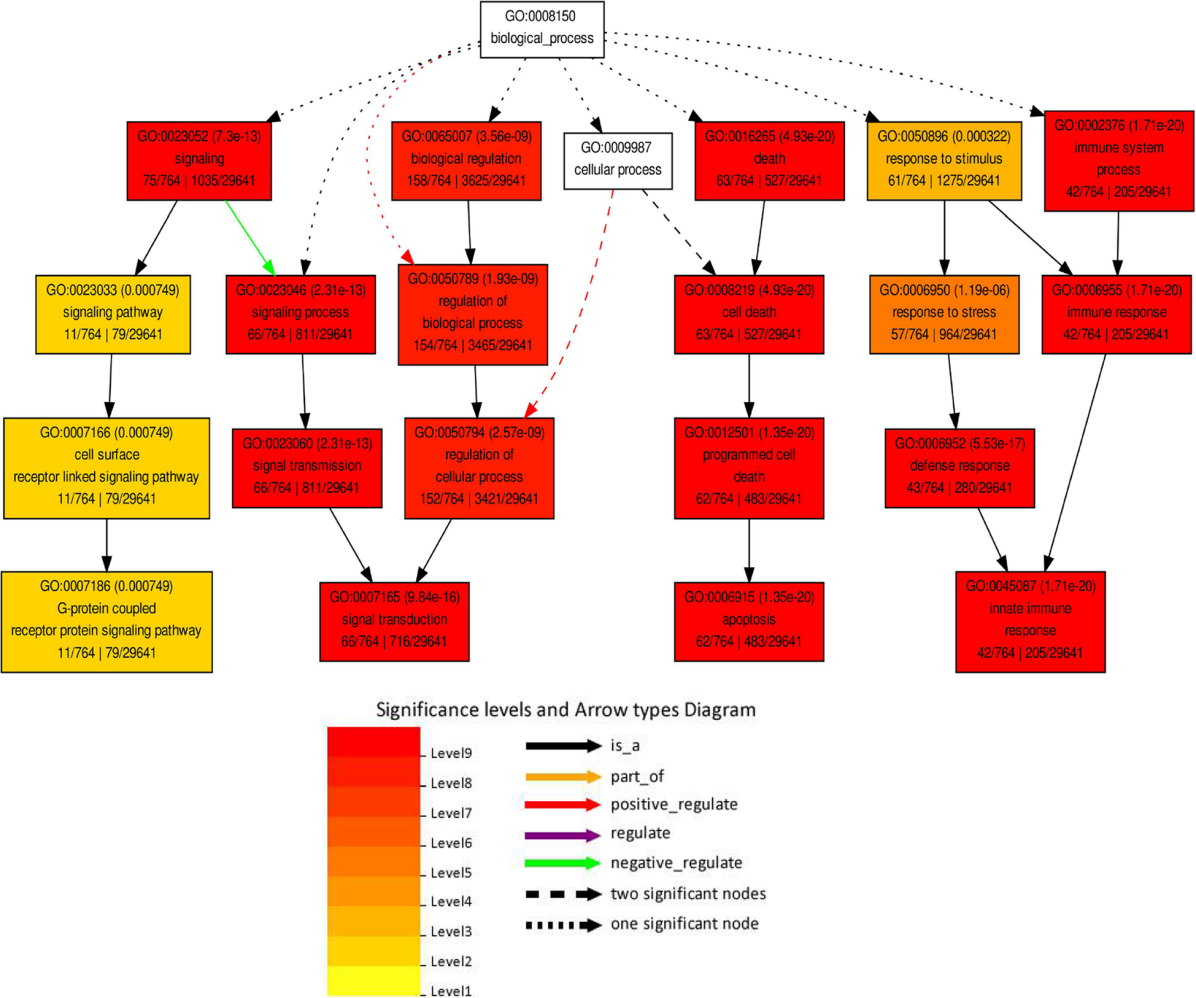
A direct acyclic graph (DAG) illustrating the biological process category generated from the Gene Ontology (GO) annotation of the predicted target miRNAs. The nodes in the image are classified into ten levels, which are associated with corresponding specific colors. The smaller of the term’s adjusted *p*-value, the more significant statistically, and the node’s color is darker and redder. Inside the box of the significant terms, the information include: GO term, adjusted p-value, GO description, item number mapping the GO in the query list and background, and total number of the query list and background. Different arrow types are also shown in the annotation diagram

To further explore the role of circRNAs in CMS in soybean, the 83 binding miRNAs of differentially expressed circRNAs were used to predict their possible target mRNAs by psRobot with the default parameters. In total, 1166 target mRNAs were predicted and further used for functional analysis (Additional file 8). A direct acyclic graph (DAG) of biological processes was obtained using the agriGO online server (Fig. 7). The DAG can indicate submitted terms and the inter-relationships between terms. From this figure, we could identify several pathways with significant enrichment, especially those involved in signal transduction and programmed cell death (PCD). A previous study showed that pollen development depended on the interaction of multiple signaling pathways, in which calmodulin was a key element [64]. Disruption of signaling pathways might cause abnormal pollen development, resulting in male sterility in soybean. In addition, PCD in plants is a cellular process similar to apoptosis, which contains fragmentation of nuclear DNA and is controlled by mitochondrion-driven signals [3]. Plant PCD plays a role in development processes such as senescence, seed germination, organ development, root tip elongation, xylem and aerenchyma formation, and disease resistance [65]. The development of plant male gametophytes in anthers requires cooperative interactions between sporophytic (anther wall) and gametophytic (microspore) cells as well as proper PCD-controlled cellular degeneration of the tapetum and the anther wall tissue [66]. Therefore, premature or delayed PCD leads to abnormality of pollen development and tapetal function, and even male sterility [67–69]. For example, the PET1-CMS cytoplasm in sunflower causes premature PCD of the tapetal cells, which then leads to abnormal anther development [70]. The KEGG pathway analysis was also used to further explore the function of predicted target mRNAs of differentially expressed circRNAs. The target mRNAs were significantly enriched in six pathways: beta-Alanine metabolism, fatty acid degradation, lysine degradation, ascorbate and aldarate metabolism, limonene and pinene degradation, and mRNA surveillance pathway (Table 3). Several studies have shown that in most plant species, the contents of amino acids, proteins, and soluble sugars in male sterile lines were lower than those in their maintainer lines [71, 72]. In this study, the pathways of amino acid degradation and material metabolism may be important causes of CMS in soybean. GO categories and KEGG pathway analyses showed that a large number of target mRNAs were involved in pollen development and male fertility, which implied that the differentially expressed circRNAs might play an important role in CMS of soybean.

**Table 3:** KEGG pathway enrichment of target mRNAs of differentially expressed circRNAs in soybean

Pathway ID	Description	Gene count	*P*-value
gmx00410	beta-Alanine metabolism	9	0.004
gmx00071	Fatty acid degradation	7	0.027
gmx00310	Lysine degradation	5	0.030
gmx00053	Ascorbate and aldarate metabolism	6	0.032
gmx00903	Limonene and pinene degradation	3	0.034
gmx03015	mRNA surveillance pathway	12	0.045

Pathway with the threshold of *P*-value < 0.05 was listed

### Prediction of protein-coding potential of soybean circRNAs

Recent studies have demonstrated that some circRNAs can be translated into polypeptides or proteins by translation initiation element internal ribosome entry site (IRES) [27]. To verify whether soybean circRNAs have a similar function, we predicted the protein-coding potential by blasting sequences of the circRNAs to all the IRES sequences at the website. A total of 165 soybean circRNAs contained at least one IRES element and an open reading frame (Additional file 9), which might have protein-coding potential. Furthermore, conserved domains of the possible protein-coding products were predicted by Conserved Domain Database (Additional file 10) [73], which might have important functions. Recent studies have shown that the protein-coding products of circRNAs can influence the function of their parental genes by interacting directly or indirectly with them [74, 75]. In this study, some parental genes of circRNAs with protein-coding potential were associated with flower and pollen development. For example, gma-circRNA0736 was predicted to encode an 81- amino acid protein, and its parental gene was a homolog of VERNALIZATION INDEPENDENCE 4 (*VIP4*) in *A. thaliana*. The *VIP4* mutants could cause slightly early flowering and variable fertility under standard growth conditions [76]. Gma-circRNA1793 was predicted to encode a 154-amino acid protein, and its parental gene was a homolog of no pollen germination related 2 (*NPGR2*) in *A. thaliana.* The *NPGR2* encodes a calmodulin-binding protein that is essential for pollen germination [77]. The protein-coding products of these circRNAs may affect flower and pollen development in soybean by affecting the function of the parental genes.

## Conclusion

In this study, a total 2867 circRNAs, of which 1009 were differentially expressed between the soybean CMS line NJCMS1A and its maintainer NJCMS1B, were identified by high-throughput deep sequencing. Tissue specific expression patterns were verified by quantitative real-time PCR. The parental genes of differentially expressed circRNAs were mainly enriched in biological processes such as metabolic process, biological regulation, and reproductive process. A large number of parental genes were related to flower development and male fertility. A total of 83 binding miRNAs were predicted among the differentially expressed circRNAs, which included well-known flower and pollen development-related miRNAs. The target mRNAs predicted for the 83 binding miRNAs were significantly enriched in signal transduction and programmed cell death. A total of 165 soybean circRNAs contained at least one IRES element and an open reading frame (ORF), indicating their potential to encode polypeptides or proteins. Our study indicated that circRNAs might participate in the regulation of flower and pollen development, which could provide a new insight into the molecular mechanisms of CMS in soybean.

## Additional files

Additional file 1: **Table S1.** Divergent primers for validation of 12 randomly-selected differentially expressed circRNAs and qRT-PCR. (XLSX 10 kb)

Additional file 2: **Table S2.** The circRNAs identified in NJCMS1A and NJCMS1B. (XLSX 254 kb)

Additional file 3: **Table S3.** The homologous circRNAs identified in this study vs. previous study of Zhao et al. (XLSX 3455 kb)

Additional file 4: **Figure S1.** Venn diagram shows the number of tissue preferentially expressed circRNAs in different tissues of soybean. (PDF 93 kb)

Additional file 5: **Table S4–1.** The up-expressed circRNAs identified in NJCMS1A and NJCMS1B; **Table S4–2.** The down-expressed circRNAs identified in NJCMS1A and NJCMS1B. (XLSX 106 kb)

Additional file 6: **Figure S2.** Junction sites were confirmed by Sanger sequencing. (PDF 2526 kb)

Additional file 7: **Table S5.** KEGG pathway enrichment of parental genes of differentially expression circRNAs in soybean. (XLSX 9 kb)

Additional file 8: **Table S6.** Predicted circRNA-miRNA-mRNA connection for differentially expressed circRNAs in NJCMS1A and NJCMS1B. (XLSX 48 kb)

Additional file 9: **Table S7.** CircRNAs with protein-coding potential. (XLSX 42 kb)

Additional file 10: **Table S8.** Conserved domains of the predicted protein-coding products. (XLSX 72 kb)

## Abbreviations

*AP2*: APETALA2
circRNA: circular RNA
CMS: Cytoplasmic male sterility
DAG: Direct acyclic graph
*E3*: dihydrolipoyl dehydrogenase
GO: Gene Ontology
*GSL10*: Glucan Synthase-Like 10
*GSL8*: Glucan Synthase-Like 8
IRES: Internal ribosome entry site
KEGG: Kyoto Encyclopedia of Genes and Genomes
m6A: N6-methyladenosine
*MEE18*: Maternal effect embryo arrest 18
miRNA: microRNA
*NADP-ICDH*: NADP-dependent isocitrate dehydrogenase
*NPGR2*: No pollen germination related 2
PCD: Programmed cell death
qRT-PCR: Quantitative real time PCR
ROS: Reactive oxygen species
*SPL*: Squamosa promoter-binding protein-like
*SQE1*: Squalene epoxidase 1
*VIP4*: VERNALIZATION INDEPENDENCE 4
WEGO: Web Gene Ontology Annotation Plot
*XRI1*: X-ray induced 1
