# Supplementary material for: Comparative analysis of circular RNAs between soybean cytoplasmic male-sterile line NJCMS1A and its maintainer NJCMS1B by high-throughput sequencing
Source: BMC Genomics. 2018 Sep 12;19:663. doi: 10.1186/s12864-018-5054-6 (PMC6134632; doi:10.1186/s12864-018-5054-6)
Supplement: Supplementary file 4 — Figure S1. Venn diagram shows the number of tissue preferentially expressed circRNAs in different tissues of soybean. (PDF 93 kb) [file 12864_2018_5054_MOESM4_ESM.pdf]

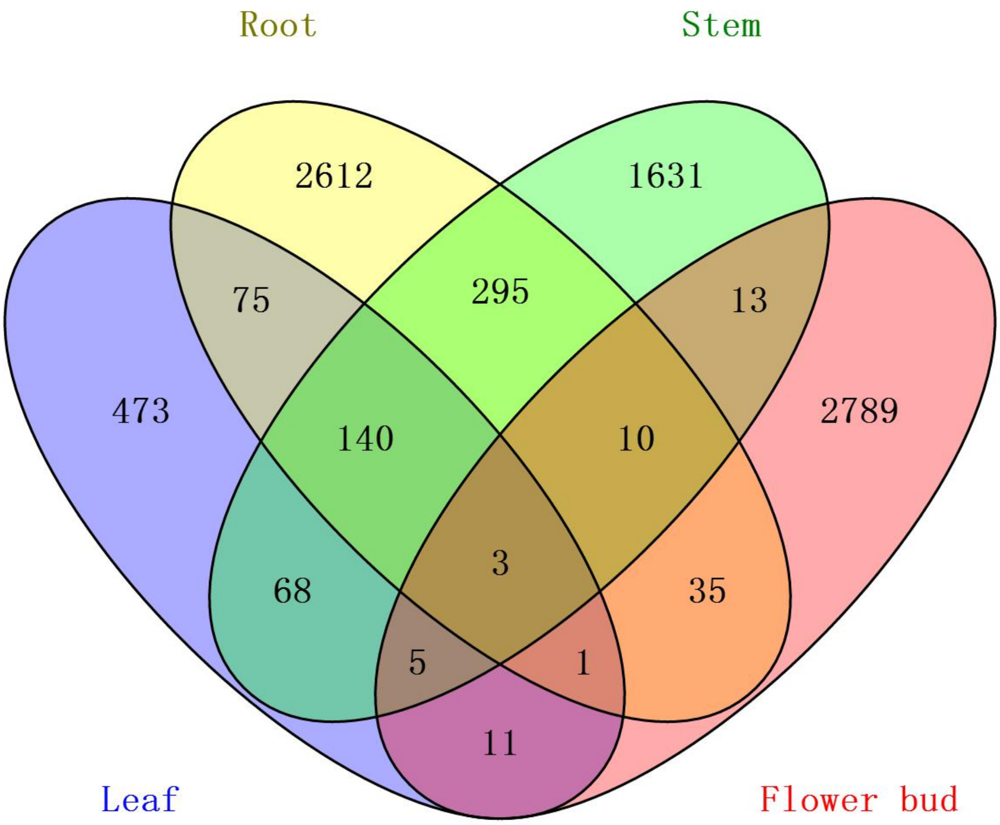

Figure S1 Venn diagram shows the number of tissue-preferentially expressed circRNAs in different tissues of soybean. The circRNAs in stems, roots and leaves of soybean were identified by Zhao et al., and the circRNAs in flower buds were identified in this study.
