# Supplementary figures and images for: Comparative analysis of circular RNAs between soybean cytoplasmic male-sterile line NJCMS1A and its maintainer NJCMS1B by high-throughput sequencing
Source: BMC Genomics. 2018 Sep 12;19:663. doi: 10.1186/s12864-018-5054-6 (PMC6134632; doi:10.1186/s12864-018-5054-6)

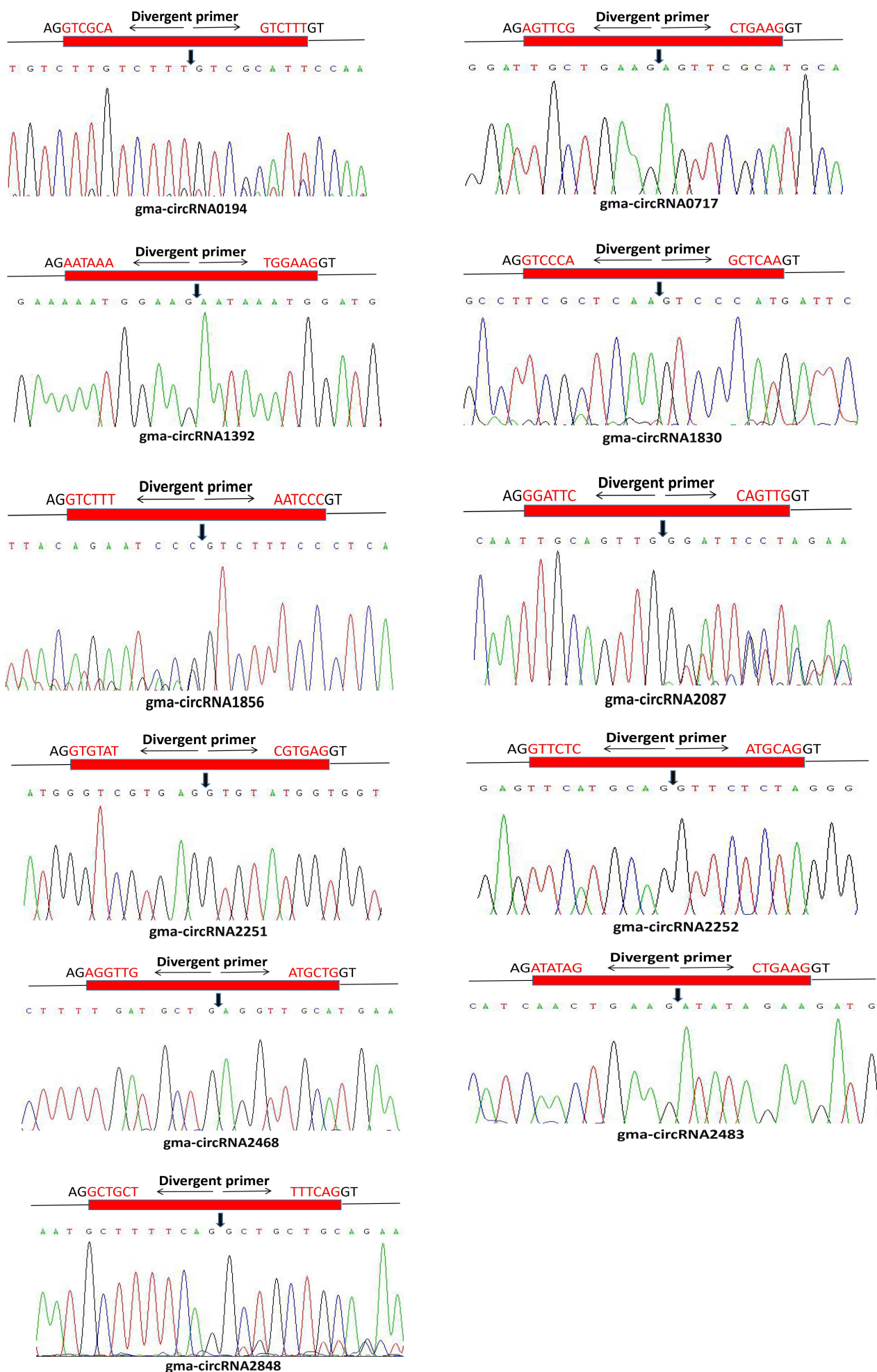

**Figure S2**

Junction sites were confirmed by Sanger sequencing.

Supplement: Supplementary file 6 — Figure S2. Junction sites were confirmed by Sanger sequencing. (PDF 2526 kb) [file 12864_2018_5054_MOESM6_ESM.pdf]
